# Awareness about Neonatal Lactose Intolerance among Chinese Neonatologists in Outpatient Settings: A Multi-Center Survey

**DOI:** 10.3390/children11081014

**Published:** 2024-08-20

**Authors:** Zhengli Wang, Liting Liu, Chao Yu, Wenyan Tang, Xiangping Ding, Xiangwen Hu, Yuan Shi

**Affiliations:** 1 Department of Neonatology, Children’s Hospital of Chongqing Medical University, Chongqing 400014, China; wangzhengli@cqmu.edu.cn (Z.W.); 481068@hospital.cqmu.edu.cn (L.L.); 2 National Clinical Research Center for Child Health and Disorders, Chongqing 400014, China; 3 Ministry of Education Key Laboratory of Child Development and Disorders, Chongqing 400014, China; 4 Chongqing Key Laboratory of Child Rare Diseases in Infection and Immunity, Chongqing 400014, China; 5 Jiangxi Hospital, Children’s Hospital of Chongqing Medical University, Nanchang 330000, China; 13576968837@139.com (W.T.); dingxiangping@sina.com (X.D.); sam850914@163.com (X.H.); 6 The First Affiliated Hospital of Chongqing Medical and Pharmaceutical College, Chongqing 400016, China; xinnuanxiangyang11@163.com

**Keywords:** awareness, neonatal lactose intolerance, neonatologists

## Abstract

Background: This study aimed to identify the specific areas of knowledge gaps regarding lactose intolerance among neonatologists in Chinese outpatient settings as well as to assess the availability of lactose intolerance testing in hospitals. Methods: A total of 278 neonatologists in outpatient settings from 144 hospitals were surveyed. To explore the awareness level, diagnosis, and treatment of neonatal lactose intolerance among neonatologists in outpatient settings, a multicenter cross-sectional survey was designed. Descriptive analysis based on frequency and percent distribution was performed for all variables. Results: Most respondents were senior doctors (256, 92.09%) from general hospitals and maternity/maternal and child health hospitals, had over 10 years of experience, and were dominantly associate chief physicians and chief physicians (211, 75.90%). A significant proportion of the participants (236, 84.89%) believed that neonatal lactose intolerance tends to be overlooked during clinical practice. When the most common symptoms of neonatal lactose intolerance were surveyed, diarrhea was selected by 142 (51.08%) respondents, followed by bloating and milk regurgitation or emesis (71, 25.54%). Other symptoms included unexplained crying (36, 12.85%), stool with milk flap or foam (15, 5.40%), and increased venting (14, 5.04%). Furthermore, the survey results indicated that the most common method for diagnosing neonatal lactose intolerance in the respondents’ hospitals was qualitative test for urinary galactose (78, 28.06%). Of the respondents, 137 (49.28%) stated that their hospital could not test for lactose intolerance. For treating lactose intolerance, the neonatologists primarily opted for exogenous lactase rather than lactose-free formula milk. Conclusions: This study sheds light on Chinese neonatologists’ awareness of neonatal lactose intolerance, revealing some knowledge gaps. The expeditious popularization and conduct of lactose intolerance-related examinations in hospitals will have a positive stimulative effect on the management of lactose intolerance in newborns.

## 1. Introduction

Neonatal lactose intolerance refers to the deficiency or reduced activity of lactase in the neonatal digestive system, which results in impaired lactose digestion and absorption [1]. The symptoms include diarrhea, abdominal distension, increased bowel sounds, intestinal colic, constipation, and vomiting [2,3]. The manifestations of this condition are varied and lack specificity. Prolonged untreated neonatal lactose intolerance can lead to chronic diarrhea, malnutrition, anemia, osteoporosis, and other long-term risks in newborns [4,5]. Thus, early detection and appropriate intervention are crucial. Currently, available treatment options encompass lactase supplementation and a lactose-free/low-lactose diet [6,7]. Nonetheless, established guidelines are not available for the diagnosis and treatment of neonatal lactose intolerance, particularly in preterm infants [1,8]. At present, it is widely accepted that if an infant is breastfed, breastfeeding should not be discontinued because of lactose intolerance; instead, exogenous lactase supplementation should be considered [9]. Several studies have documented substantial improvement in gastrointestinal symptoms, such as diarrhea, bloating, nausea, and abdominal pain, after oral lactase administration [10,11,12].

Unfortunately, knowledge of neonatal lactose intolerance has not been adequately disseminated, and as a result, many patients remain undiagnosed [13]. In China, neonatologists often encounter cases of neonatal lactose intolerance in outpatient clinics, thereby providing them with a valuable opportunity for prevention and control efforts. Although there is a lack of consensus on specific related aspects. On the one hand, it is imperative to educate parents about lactose intolerance via various means. On the other hand, neonatologists need to augment their understanding [14]. Regrettably, there are no reports on the level of awareness or treatment of lactose intolerance among neonatal outpatient doctors in China.

The level of understanding and awareness among neonatologists regarding the diagnosis and treatment of lactose intolerance, as well as the availability of lactose intolerance testing, may significantly impact the timely management of this condition in newborns. Furthermore, it has come to our attention that several hospitals in China do not offer lactose intolerance testing. Therefore, we hope to identify the specific areas of knowledge gaps regarding lactose intolerance among neonatal doctors as well as to assess the availability of lactose intolerance testing in hospitals. This approach is expected to provide targeted education to neonatologists and advocate the importance of incorporating lactose intolerance testing in healthcare facilities.

## 2. Materials and Methods

### 2.1. Study Design and Population

The study was a multicenter online survey conducted between 22 December 2023 and 30 January 2024. We randomly selected 278 neonatologists in outpatient settings from 144 hospitals located in 62 cities spanning 23 provinces and municipalities of China to participate in the survey. We launched this electronic questionnaire survey for neonatologists in outpatient settings through “WeChat group” or face-to-face invitation. Neonatologists in outpatient settings who volunteered to participate in the questionnaire study were included. Doctors from other specialties and neonatologists who did not agree to participate in the survey were excluded. The interviewed neonatologists were assured that the results of the survey would be used only for scientific research, but they were not informed of the specific research contents. The respondents remained anonymous. The study protocol was approved by the institutional ethics committee of the Children’s Hospital of Chongqing Medical University.

### 2.2. Questionnaires and Data Collection

After conducting a comprehensive literature review and consulting experienced neonatologists, a questionnaire containing 22 questions was developed to understand the knowledge level of neonatologists about neonatal lactose intolerance. The questionnaire was categorized into the following three parts: (1) personal information, including the hospital level, sex, years of working, and professional title of the respondents; (2) awareness of the respondents about neonatal lactose intolerance, including its clinical manifestations, diagnostic methods, and treatment options; and (3) knowledge of the respondents about lactase. The questionnaire was created using the “Wenjuanxing” platform (Changsha Ranxing Information Technology Co., Ltd., Changsha, China). The respondents completed the questionnaire directly on their mobile devices. Before participating in the survey, the study was explained to them, including the sentence “By completing the questionnaire, you are consenting to participate”. Therefore, the respondents who completed the questionnaire were considered to have provided their consent.

### 2.3. Data Analysis

The data were reviewed separately by members of the research team and were entered into an MS Office Excel file. Descriptive analysis based on frequency and percent distribution was performed for all variables, including data on the participants’ demographic profiles and awareness of lactose intolerance disorder and lactase enzyme. Data processing and analysis were performed using R version 4.3.0 (21 April 2023), along with Zstats v0.90 (https://www.medsta.cn/software, accessed on 21 April 2023). For categorical variables, the proper statistical tests were applied, including the Chi-squared test and Fischer’s exact test. In addition, *p* < 0.05 was considered to indicate statistical significance.

## 3. Results

### 3.1. Demographic Data

The respondents were predominantly senior physicians from tertiary hospitals, and the majority of them had been working for more than 10 years. The detailed characteristics of the respondents are presented in Table 1.

### 3.2. The Majority of the Respondents of the Respondents Self-Assessed Their Awareness of Neonatal Lactose Intolerance as “Partial Understanding” and Deemed It Prone to Being Ignored in Clinical Work

Among these respondents, 78 (28.06%) were very much aware of neonatal lactose intolerance, 169 (60.79%) were somewhat aware of this condition, and only 1 (0.36%) was not very much aware of it. More than half of the participants (160, 57.55%) indicated that neonatal lactose intolerance accounted for <20% of neonatal outpatients. Approximately one-third of the participants (98, 35.25%) stated that neonatal lactose intolerance accounted for approximately 20–39.99% of neonatal outpatients. The respondents agreed that this disease was more common in preterm infants. Most participants (236, 84.89%) believed that neonatal lactose intolerance tends to be overlooked during clinical practice. When they encountered newborns with diarrhea, bloating, emesis, or unexplained crying, 59 (21.22%) neonatologists stated that they routinely tested for lactose intolerance, whereas 219 (78.78%) did not. These results are given in Table 2.

### 3.3. The Majority of the Respondents Opined That Neonatal Lactose Intolerance Is Predominantly Developmental Lactose Intolerance with Diarrhea as the Principal Manifestation

When the etiology of neonatal lactose intolerance was surveyed, it was reported to be developmental lactose intolerance by 246 (88.49%) respondents. Furthermore, when the most common symptoms of neonatal lactose intolerance were inquired, diarrhea was selected by 142 (51.08%) respondents (Table 3).

### 3.4. The Majority of the Respondents Gave Priority to Lactase for the Treatment of Cases of Neonatal Lactose Intolerance

The survey results demonstrated that the most common method for diagnosing neonatal lactose intolerance in the hospitals of the respondents was qualitative testing for urinary galactose (78, 28.06%). About half of the respondents (49.28%) mentioned that their hospital did not test for lactose intolerance. Whenever newborns exhibited lactose intolerance and significant gastrointestinal symptoms, most respondents preferred exogenous lactase supplementation over switching to lactose-free formula milk as an adjunct to conventional symptomatic medications. The findings are elaborated in Table 4.

### 3.5. The Majority of the Respondents Self-Assessed Their Awareness of Neonatal Lactase Usage in Terms of Indications, Methods, and Treatment Options as “Partial Understanding”

The survey results revealed that most respondents selected “completely understand” or “partly understand” for the question on their awareness of indications, methods, and treatment options for the use of lactase, and only a few respondents indicated a limited understanding. Moreover, 96 (34.53%) respondents stated that prophylactic lactase supplementation can be given to newborns at high risk of lactose intolerance, and 67 (24.1%) respondents indicated that supplementation of lactase should only be administered to newborns diagnosed with lactose intolerance. In addition, 93.88% reported that liquid lactase is suitable for newborns with lactose intolerance. These results are presented in a detailed manner in Table 5.

### 3.6. More Neonatologists from the Children’s Hospitals Believed That the Duration of the Use of Lactase Was “≥2 Weeks and Symptoms Improved”

No significant differences were noted in the baseline characteristics of neonatologists from three different types of hospitals (i.e., general hospitals, maternity/maternal and child health hospitals, and children’s hospitals), and they were majorly aware of neonatal lactose intolerance. However, their understanding of the duration of lactase use was inconsistent, and more neonatologists from the children’s hospitals believed that the duration of the use of lactase was more than 2 weeks when compared to that of the other two types of hospitals (*p* = 0.016; Table 6).

## 4. Discussion

The prevalence of neonatal lactose intolerance in China is approximately 40% and accounts for 12–30% of all children with lactose intolerance [15]. The predominant symptoms of this condition are diarrhea, abdominal distension, increased exhaustion, and colic [2]. However, in a small number of patients, abdominal distension or colic is the only clinical manifestation, and it is difficult to diagnose them; hence, the rate of missed diagnosis is likely to be high. Therefore, this survey aimed to comprehensively assess the awareness status of neonatal lactose intolerance among Chinese neonatologists.

The respondents primarily hailed from general hospitals, maternal and child health hospitals, and children’s hospitals. Most of them held professional titles as chief physicians or associate chief physicians. Although there were certain differences in the views, the overall awareness of the diagnosis and treatment of neonatal lactose intolerance was generally unified. According to a study, lactose intolerance can be categorized into four types: primary lactase deficiency, secondary lactase deficiency, developmental lactase deficiency, and congenital lactase deficiency [16]. Neonatal lactose intolerance is chiefly characterized by developmental and secondary lactase deficiency. Developmental lactase deficiency, also known as relative lactase deficiency, is more common in premature infants because lactase and other disaccharides in the gastrointestinal tract begin to develop rapidly after only 34 weeks of gestation and peak at 40 weeks [17]. This finding is consistent with the opinion of >50% of the respondents that lactose intolerance is more common in preterm infants.

Up to 70% of the global population exhibits lactase non-persistence, but not all individuals are intolerant to lactose due to various nutritional and genetic factors that influence tolerance [18]. Lactose intolerance is characterized by a range of symptoms, such as abdominal pain, bloating, excessive gas, diarrhea, audible bowel sounds, and, occasionally, nausea and vomiting [6]. In rare instances, lactose intolerance can decrease gastrointestinal motility, potentially causing constipation, which may be linked to methane production [19]. In addition to gastrointestinal symptoms, neonatal lactose intolerance can also result in extra-gastrointestinal symptoms, such as oral ulcers, arrhythmias, and eczema [1]. In severe cases, it may also lead to malnutrition, growth retardation, anemia, and abnormal bone metabolism [7]. However, in our survey, several respondents missed these symptoms. This result serves as a reminder to pay more attention to the diverse range of clinical symptoms associated with lactose intolerance in newborns.

The understanding of lactose intolerance varies among different doctors. An international cross-sectional survey focusing on medical professionals for the management of milk protein allergy and lactose intolerance in infants and young children reported that the respondents were confident in managing lactose intolerance; however, there were significant knowledge disparities regarding the differences between food protein allergy and lactose intolerance, as well as marked differences in the level of understanding of relevant clinical practice guidelines [20]. A nationwide survey conducted in Spain compared the knowledge and clinical management of primary care physicians (PCPs) with that of Spanish gastroenterologists (GEs) and discovered that the level of understanding of lactose intolerance was similar. Nevertheless, a higher proportion of PCPs lacked epidemiological awareness (*p* < 0.01). GEs tended to regard lactose intolerance as a “minor” condition, and the symptoms perceived as suspicious for lactose intolerance were alike in both groups [21]. Although most of the surveyed neonatologists self-assessed that they had a good understanding of lactose intolerance, some respondents had an inadequate awareness of the symptoms, clinical signs, and diagnosis of the disease, which is a key finding of this study. The survey results of this questionnaire are self-assessments, and there may be situations of non-objectivity. Therefore, the further reinforcement of the professional knowledge education for neonatologists and the augmentation of their understanding of the clinical practice guidelines pertaining to the diagnosis and treatment of lactose intolerance might potentially facilitate the enhancement of the clinical outcomes of the pediatric patients.

The examination methods for diagnosing neonatal lactose intolerance include the following: hydrogen breath test, lactose tolerance test, reducing sugar and pH determination in the stool sample, qualitative test for urinary galactose, jejunum mucosal biopsy, and genetic test [22]. The questionnaire survey revealed that reducing sugar and pH determination in the stool sample and qualitative test for urinary galactose were the most common methods for determining lactose intolerance in the hospitals of the respondents. The method of fecal reducing sugar and pH determination is noninvasive, simple, and suitable for neonates. As lactase activity is not completely lost in patients with developmental lactase deficiency, qualitative tests for urinary galactose may yield false negative results. Nonetheless, almost half of the respondents stated that their hospital did not perform tests for diagnosing neonatal lactose intolerance, which is a crucial factor limiting its clinical diagnosis. Inadequate awareness of the manifestations and diagnosis of the condition may prevent neonatologists from providing reasonable advice. However, the sensitivity and specificity of these tests are affected by factors such as reagents, testing methods, and samples, making them unstable [8]. Consequently, relying solely on test results to reach a conclusion may contribute to missed diagnoses [23]. In clinical practice, diagnosing lactose intolerance in newborns involves not only laboratory testing, but also diagnostic treatment based on an initial assessment of clinical symptoms to observe whether the clinical symptoms are alleviated [24].

The management of neonatal lactose intolerance includes approaches such as low-lactose/lactose-free formula milk and lactase supplementation. However, a long-term lactose-free diet prevents the absorption of calcium, iron, and zinc in newborns and may affect their nervous system development [25]. Neonatologists still need to enhance the detailed elaboration to parents on the relevant knowledge regarding the treatment and follow-up of lactose intolerance in order to reduce unnecessary feeding with lactose-free milk powder. A survey on parenting practices among Chinese in Singapore reported that half of the respondents thought that it was necessary to switch to lactose-free formula once a child developed diarrhea, and only 44% felt that pediatricians allocated sufficient time to discuss parenting issues [26]. Moreover, breast milk offers numerous advantages and plays a vital role in the growth and immune regulation of neonates. According to the European Society for Pediatric Gastroenterology, Hepatology, and Nutrition/European Society for Pediatric Infectious Diseases evidence-based guidelines for the management of acute gastroenteritis in children, breastfed infants should be exclusively fed with human milk in all cases [27]. Given the increasing emphasis on breastfeeding, the use of lactose-free formula milk as a substitute for breast milk may not be an ideal choice. Several studies have reported that lactase supplementation can alleviate the symptoms associated with neonatal lactose intolerance, such as reducing the duration of diarrhea and relieving intestinal colic [28,29,30]. Lactase supplementation can enhance weight gain in preterm infants without altering the original dietary structure [11], thus making it an efficacious remedy for neonatal lactose intolerance.

This questionnaire survey revealed that neonatologists from general hospitals, maternity/maternal and child health hospitals, and children’s hospitals shared the same awareness level about neonatal lactose intolerance. More than 90% of neonatologists, especially those from children’s hospitals, opted for exogenous lactase to treat lactose intolerance. A greater proportion of neonatologists from children’s hospitals believed that the duration of treatment was inclined to be ≥2 weeks when compared to the other two types of hospitals. However, proper guidelines on the course and dosage of lactase supplementation are still lacking, and further prospective, multicenter, large-sample clinical studies are needed to validate our findings.

Nonetheless, there are certain limitations in this study. Owing to resource limitations, a strict sampling survey and a large sample survey could not be conducted. The source and number of respondents in this survey were restricted and mainly comprised clinicians with senior professional titles, extensive work experience, and employment in large tertiary hospitals. All respondents voluntarily participated in the survey, and the number of participants from each unit was small. Some of the questions are subjective and may be biased from the actual situation of the respondents. Therefore, the awareness level of Chinese neonatologists regarding neonatal lactose intolerance might not have been completely represented.

## 5. Conclusions

This study sheds light on Chinese neonatologists’ awareness of neonatal lactose intolerance, revealing some knowledge gaps. The expeditious popularization and conduct of lactose intolerance-related examinations in hospitals will have a positive stimulative effect on the management of lactose intolerance in newborns.

## Figures and Tables

**Table 1:** Personal information about the respondents (*n* = 278).

Variable	*n*. (%)
Gender	
Female	204 (73.38)
Male	74 (26.62)
Type of hospital	
General hospitals	130 (46.76)
Maternity/maternal and child health hospitals	97 (34.89)
Children’s hospitals	51 (18.35)
Hospital level	
Tertiary	266 (95.68)
Secondary	9 (3.24)
Other	3 (1.08)
The number of patients in the neonatal unit	
<20	11 (3.96)
20–49	117 (42.09)
50–99	73 (26.26)
100–199	59 (21.22)
≥200	18 (6.47)
Professional titles	
Senior	211 (75.90)
Intermediate	64 (23.02)
Primary	3 (1.08)
Years of working	
<5	4 (1.44)
5–9	18 (6.47)
10–19	101 (36.33)
≥20	155 (55.76)

**Table 2:** Overall status of awareness about neonatal lactose intolerance and some information about this disease in their hospitals (*n* = 278).

Variable	*n*. (%)
Awareness about neonatal lactose intolerance	
Complete understanding	78 (28.06)
Partial understanding	169 (60.79)
Preliminary understanding	30 (10.79)
A limited understanding	1 (0.36)
Incomprehension	0 (0)
The percentage of neonatal lactose intolerance patients in outpatient clinics (%)	
<20	160 (57.55)
20–39.99	98 (35.25)
40–59.99	16 (5.76)
≥60	4 (1.44)
Is neonatal lactose intolerance more common in full-term or preterm infants?	
Full-term infants	31 (11.15)
Preterm infants	155 (55.76)
Full-term and preterm infants are equal	92 (33.09)
Do you think neonatal lactose intolerance is easy to ignore in clinical work?	
Yes	236 (84.89)
No	42 (15.11)
If you encountered newborns with diarrhea, bloating, emesis, or unexplained crying, would you test for lactose intolerance?	
Yes	59 (21.22)
No	219 (78.78)

**Table 3:** Status of awareness about neonatal lactose intolerance etiology and symptoms (*n* = 278).

Variable	*n*. (%)
Awareness of the etiology of neonatal lactose intolerance	
Developmental lactose intolerance	246 (88.49)
Secondary lactase intolerance	29 (10.43)
Drug factors	2 (0.72)
Intestinal inflammation	1 (0.36)
The most common symptoms of neonatal lactose intolerance	
Diarrhea	142 (51.08)
Bloating, milk regurgitation, or emesis	71 (25.54)
Unexplained crying	36 (12.85)
Stool with milk flap or foam	15 (5.40)
Increased venting	14 (5.04)
Other symptoms of neonatal lactose intolerance (more than one answer is acceptable)	
Chronic diarrhea and severe bloody stool	252 (90.65)
Malnutrition	269 (96.76)
Anemia	149 (53.60)
Abnormal bone metabolism	139 (50.00)
Intestinal colic	259 (93.17)
Oral ulcers, arrhythmias, and eczema	74 (26.62)

**Table 4:** Status of awareness about neonatal lactose intolerance diagnoses and treatments (*n* = 278).

Variable	*n*. (%)
The most common method for diagnostic neonatal lactose intolerance in your hospital (more than one answer is acceptable)	
Hydrogen breath test	29 (10.43)
Lactose tolerance test	30 (10.79)
Reducing sugar and pH determination of stool	71 (25.54)
Qualitative test for urinary galactose	78 (28.06)
Jejunum mucosal biopsy	13 (4.68)
Genetic test	22 (7.91)
Could not test	137 (49.28)
When lactose intolerance occurs in newborns, which method do you prefer to use in addition to the conventional symptomatic drugs?	
Exogenous lactase	263 (94.60)
Lactose-free formula	14 (5.04)
Other	1 (0.36)
When neonatologists encounter neonates with diarrhea, abdominal distension, or unexplained crying that does not respond well to conventional treatment, lactase can be added as an adjunctive therapy	
No	1 (0.36)
Yes	253 (91.01)
Not sure. I’ll decide after further examination	24 (8.63)

**Table 5:** Status of awareness about lactase (*n* = 278).

Variable	*n*. (%)
Awareness of indications for the use of lactase	
Complete understanding	64 (23.02)
Partial understanding	174 (62.59)
Preliminary understanding	38 (13.67)
A limited understanding	2 (0.72)
Incomprehension	0 (0)
Awareness of methods and treatment for the use of lactase	
Complete understanding	72 (25.90)
Partial understanding	164 (58.99)
Preliminary understanding	38 (13.67)
A limited understanding	4 (1.44)
Incomprehension	0 (0)
How long the lactase is used in newborns diagnosed with secondary lactase intolerance?	
3–5 days	7 (2.52)
5–7 days	29 (10.43)
More than 2 weeks and symptoms improved	242 (87.05)
Do you give prophylactic supplementation of lactase to newborns at high risk of lactose intolerance?	
Frequently	96 (34.53)
Occasionally	115 (41.37)
Supplementation of lactase are only given to newborns diagnosed with lactose intolerance	67 (24.10)
Which lactase do you think is more suitable for lactose-intolerant newborns?	
Liquid	261 (93.88)
Solid	6 (2.16)
Both are fine	11 (3.96)

**Table 6:** Comparison of awareness about neonatal lactose intolerance among neonatologists in three different types of hospitals.

Items	General Hospitals (*n* = 130)	Maternity/Maternal and Child Health Hospitals (*n* = 97)	Children’s Hospitals (*n* = 51)	*p*
Males, *n* (%)	32 (24.62)	30 (30.93)	12 (23.53)	0.487
Intermediate and senior titles, *n* (%)	90 (69.23)	79 (81.44)	42 (82.35)	0.051
Works in a tertiary hospital, *n* (%)	126 (96.29)	91 (93.81)	49 (96.08)	0.443 *
Their hospital can test for lactose intolerance, *n* (%)	70 (53.85)	45 (46.39)	26 (50.98)	0.539
Completely understand the etiology of neonatal lactose intolerance, *n* (%)	120 (92.31)	83 (85.57)	43 (84.31)	0.291 *
It is believed that newborns with diarrhea, bloating, emesis or unexplained crying are tested for lactose intolerance, *n* (%)	30 (23.08)	16 (16.49)	13 (25.49)	0.708
It is believed that exogenous lactase is more suitable for lactose intolerance than lactose-free formula, *n* (%)	121 (93.08)	92 (94.85)	50 (98.04)	0.726 *
Completely understand indications for the use of lactase, *n* (%)	34 (26.15)	18 (18.56)	12 (23.53)	0.498 *
It is believed that the lactase should be used for ≥2 weeks in newborns diagnosed with secondary lactase intolerance, *n* (%)	116 (89.23)	78 (80.41)	48 (94.12)	0.016 *
It is believed that liquid lactase is more suitable for newborns with lactose intolerance, *n* (%)	90 (92.78)	90 (92.78)	51 (100.00)	0.406 *

* Fisher’s exact.

## Data Availability

The datasets used and/or analyzed during the current study are available from the corresponding author upon reasonable request.
